# Identification of Important Factors Causing Developmental Arrest in Cloned Pig Embryos by Embryo Biopsy Combined with Microproteomics

**DOI:** 10.3390/ijms232415975

**Published:** 2022-12-15

**Authors:** Yuxing Zhang, Liusong Yang, Yiqian Zhang, Yalin Liang, Huaxing Zhao, Yanan Li, Gengyuan Cai, Zhenfang Wu, Zicong Li

**Affiliations:** 1 National Engineering Research Center for Breeding Swine Industry, College of Animal Science, South China Agricultural University, Guangzhou 510030, China; 2 Department of Animal Genetics, Breeding and Reproduction, College of Animal Science, Guangzhou 510030, China; 3 Guangdong Provincial Key Laboratory of Agro-Animal Genomics and Molecular Breeding, College of Animal Science, South China Agricultural University, Guangzhou 510030, China; 4 Guangdong Provincial Laboratory of Lingnan Modern Agricultural Science and Technology, Guangzhou 510642, China

**Keywords:** microproteomics, cloned pig embryos, embryo biopsy, *PDCD6*, *PLK1*

## Abstract

The technique of pig cloning holds great promise for the livestock industry, life science, and biomedicine. However, the prenatal death rate of cloned pig embryos is extremely high, resulting in a very low cloning efficiency. This limits the development and application of pig cloning. In this study, we utilized embryo biopsy combined with microproteomics to identify potential factors causing the developmental arrest in cloned pig embryos. We verified the roles of two potential regulators, *PDCD6* and *PLK1*, in cloned pig embryo development. We found that siRNA-mediated knockdown of *PDCD6* reduced mRNA and protein expression levels of the pro-apoptotic gene, *CASP3*, in cloned pig embryos. *PDCD6* knockdown also increased the cleavage rate and blastocyst rate of cloned porcine embryos. Overexpression of *PLK1* via mRNA microinjection also improved the cleavage rate of cloned pig embryos. This study provided a new strategy to identify key factors responsible for the developmental defects in cloned pig embryos. It also helped establish new methods to improve pig cloning efficiency, specifically by correcting the expression pattern of *PDCD6* and *PLK1* in cloned pig embryos.

## 1. Introduction

Somatic cell nuclear transfer (SCNT), also called cloning, is a powerful technique that can reprogram somatic cells into totipotent embryos and full-term animals. It has applications in multiplying superior livestock, rescuing endangered animal species [1,2], generating SCNT-derived embryonic stem cells for human therapy [3], producing genetically modified animals for breeding [4], pharmaceutical protein synthesis [5], or xenotransplantation [6]. Therefore, SCNT holds great promise for the livestock industry, life science, and biomedicine. However, the full-term developmental efficiency of cloned mammalian embryos is extremely low because the birth rate in pigs and mice is approximately only 1% [7] and 2% [8,9], respectively. The low success rate hinders the development and application of cloning.

The low developmental competence of cloned embryos is primarily caused by key reprogramming errors, such as the ectopic expression of Xist, aberrant DNA re-methylation, persisting histone modification, and bi-allelic expression of the imprinted genes, sfmbt2, jade1, Gab1, and smoc1 [8,10,11]. Correction of these reprogramming errors have been shown to dramatically enhance animal cloning efficiency [8,10,11].

Cloning efficiency can be improved by identifying the molecular defects that cause abnormal development in cloned embryos. Some studies have compared the gene expression and epigenetic modification profiles of cloned and fertilization-derived embryos and identified several vital reprogramming barriers in the cloned embryos [12,13,14]. Important factors that control SCNT embryo development were also discovered by comparing the DNA methylation and RNA sequencing results of arrested and normally developed SCNT embryos. This was performed using the embryo biopsy technique [15,16]. In the above-mentioned studies, various omics approaches, such as RNA sequencing, whole genome bisulfite sequencing, and Chip-seq, were employed to identify the factors regulating the developmental capacity of cloned embryos. [12,13,14,15,16]. While proteomics can also be used to analyze molecules that regulate cloning efficiency, it is difficult to obtain an adequate number of cloned embryos for regular proteomic analysis. Regular proteomics typically requires a large quantity of cells for analysis. Recently, a technique called microproteomics, also known as nanoproteomics, was successfully developed to analyze the protein profiles of samples with low cell numbers, such as early embryo samples [17,18,19,20].

To identify the factors that cause developmental defects in cloned pig embryos, this study utilized embryo biopsy combined with microproteomics to compare the proteomes between cloned porcine embryos arrested at the two-cell stage and embryos that reached the blastocyst stage. Two factors were selected from the results of our analysis and were verified to play important roles in regulating the developmental ability of cloned porcine embryos.

## 2. Results

### 2.1. Tracing the Developmental Fates of Different SCNT Embryos by Embryo Biopsy

**Figure 1:**
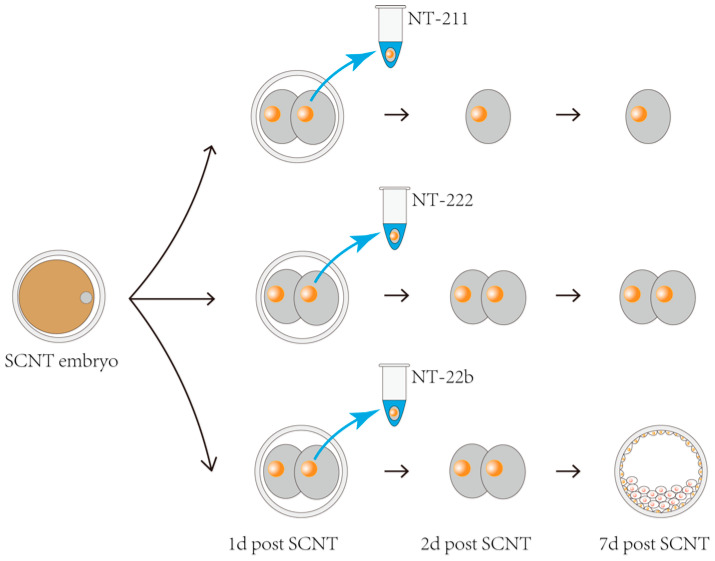
Schematic diagram of the sampling and grouping of cloned porcine embryos. One blastomere was isolated at the two-cell-stage and frozen for later microproteomic analysis. The frozen blastomeres were divided into groups NT-211, NT-222, and NT-22b according to the developmental fate of the remaining blastomeres in each embryo. The NT-211, NT-222, and NT-22b groups represent the SCNT embryos arrested at the two-cell stage (with only one cell), the SCNT embryos arrested at the four-cell stage (with two cells), and the SCNT embryos that developed to the blastocyst stage, respectively.

The gene expression profiles of cloned pig embryos show initial abnormalities at the two-cell stage, compared with that of in vivo fertilization-derived embryos [21]. Therefore, it was determined that the gene expression pattern at this stage is critical for subsequent development. To conduct microproteomic analysis, we needed to collect two-cell stage SCNT embryo samples with different developmental potentials. We generated 335 two-cell stage SCNT pig embryos and isolated one blastomere from each embryo, which were then frozen for later analysis. The remaining blastomeres in each embryo were cultured to study their actual developmental fates. During our study, we discovered that the SCNT embryos that had one blastomere removed at the two-cell stage could be divided into four groups according to their developmental fates. These four groups comprised (1) SCNT embryos arrested at the two-cell stage, (2) SCNT embryos arrested at the four-cell stage, (3) SCNT embryos that developed into blastocysts, and (4) SCNT embryos arrested at other stages (eight-cell to the morula stages) that were not easy to determine (Table 1). The frozen blastomeres corresponding to the first three groups were designated as the NT-211, NT-222, and NT-22b groups, respectively. These were subjected to microproteomic analysis. The frozen blastomeres of the fourth group were not analyzed because the embryos in this group were arrested at different stages. The schematic diagram for sampling in the first three groups is shown in Figure 1.

### 2.2. Identification of Potential Factors That Regulate the Developmental Fates of Cloned Porcine Embryos

**Figure 2:**
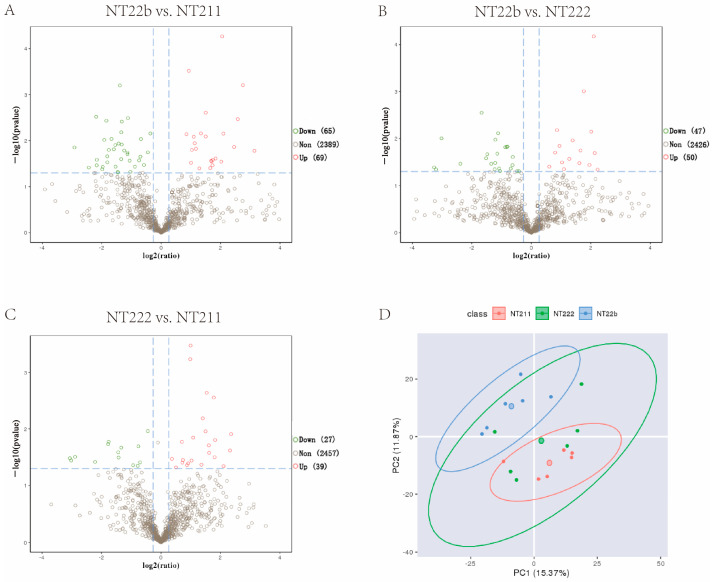
Microproteomic analysis of the three groups with different developmental fates. (**A**) Comparison of the differentially expressed proteins (DEPs) between the NT-22b and NT-211 groups. (**B**) Comparison of the DEPs between the NT-22b and NT-222 groups. (**C**) Comparison of the DEPs between the NT-222 and NT-211 groups. (**D**) Principal component analysis of the samples in the NT-211, NT-222, and NT-22b groups.

Microproteomics detected 648–1186 suspected proteins in the three groups (Table 2). We analyzed the differentially expressed proteins (DEPs) among the groups. Compared with the NT-211 group, the NT-22b group had 69 upregulated proteins and 65 downregulated proteins (Figure 2A). In comparison with the NT-22b group, the NT-222 group had 50 upregulated proteins and 47 downregulated proteins (Figure 2B). Compared with the NT-222 group, the NT-211 group had 39 upregulated proteins and 27 downregulated proteins (Figure 2C). NT-22b vs. NT-211 has more DEPs than NT-22b vs. NT-222, and NT-222 vs. NT-211. Furthermore, principal component analysis (PCA) demonstrated that the NT-211 samples were obviously separated from the NT-22b samples, whereas the NT-222 group samples were distributed between the other two groups (Figure 2D). These results suggested that the proteomic difference between NT-22b and NT-211 groups is bigger than those between NT-22b and NT-222 groups, and between NT-222 and NT-211 groups. As such, we decided to focus on the DEPs between the NT-22b and NT-211 groups because these DEPs were the most likely determine whether cloned embryos would arrest at the two-cell stage or develop to the blastocyst stage.

**Figure 3:**
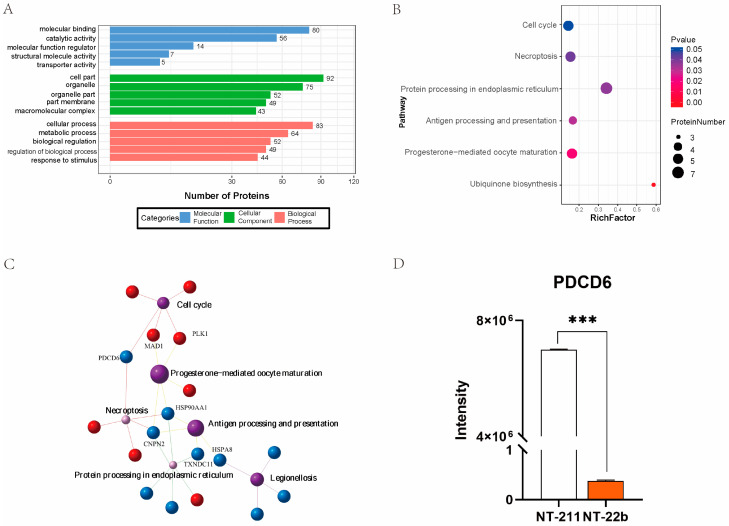
Analysis of the DEPs between the NT-22b and NT-211 groups. (**A**) Annotation analysis of the DEPs based on the Gene Ontology (GO) database. The top 5 entries in each GO category are shown. (**B**) Pathway enrichment analysis of the DEPs based on the Kyoto Encyclopedia of Genes and Genomes (KEGG) database. (**C**) KEGG pathway association network analysis (**D**) Comparison of PDCD6 protein levels between the NT-211 group and NT-22b groups. “***” represents a significant difference at *p* < 0.001.

To annotate the functions of the DEPs between the NT-22b and NT-211 groups, the DEPs were subjected to Gene Ontology (GO) enrichment analysis. GO analysis showed that the identified DEPs participated in molecular binding, catalytic activity, cell part, organelle, and cellular processes, and metabolic processes (Figure 3A). Kyoto Encyclopedia of Genes and Genomes (KEGG) pathway analysis suggested that the same DEPs were enriched in the cell cycle, necroptosis, protein processing in endoplasmic reticulum, antigen processing and presentation, progesterone-mediated oocyte maturation, and ubiquinone biosynthesis (Figure 3B). Association network analysis of the enriched KEGG pathways demonstrated that PDCD6 was involved in regulating the two most enriched KEGG pathways, the cell cycle and necroptosis pathways (Figure 3C). Of particular note, *PDCD6*, also known as apoptosis related gene-2 (ALG-2), is a regulator of apoptosis [22,23,24]. Apoptosis plays an important role in regulating the development of cloned embryos [25,26,27,28]. Moreover, the microproteomic data showed that PDCD6 protein expression level was much higher in the NT-211 group than in the NT-22b group (Figure 3D), suggesting that high PDCD6 expression is associated with the developmental arrest of NT-211 cloned embryos.

### 2.3. Microinjection of PDCD6 siRNA Significantly Improved the Developmental Efficiency of Porcine SCNT Embryos

**Figure 4:**
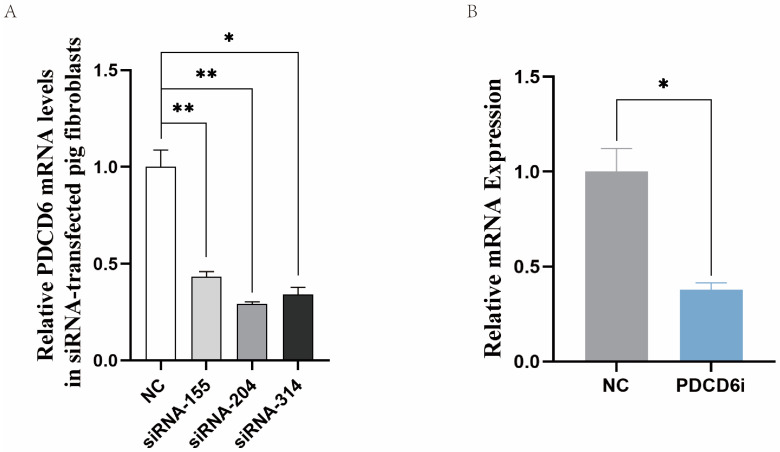
Inhibition of *PDCD6* expression in porcine fibroblasts and cloned embryos via RNA interference. (**A**) Selection of effective *PDCD6* siRNAs in porcine fibroblasts. siRNA-155, siRNA-204, and siRNA-314 are three different *PDCD6* siRNAs. (**B**) Inhibition of *PDCD6* expression in cloned pig embryos by microinjection of siRNA-204. *PDCD6*i represents *PDCD6* siRNA. Quantitative polymerase chain reaction was used to measure the expression levels of *PDCD6* at the two-cell stage of the cloned porcine embryos injected with *PDCD6* siRNA. NC, negative control siRNA. “*” and “**” represent a significant difference at *p* < 0.05 and *p* < 0.01, respectively.

To investigate whether high *PDCD6* expression was related to the arrest of the SCNT embryos in the NT-211 group, we synthesized and selected effective *PDCD6* siRNAs that could significantly reduce *PDCD6* mRNA levels in transfected porcine fibroblasts (Figure 4A). We microinjected different concentrations of *PDCD6* siRNA into cloned porcine embryos and found that 20 μM of *PDCD6* siRNA significantly inhibited *PDCD6* mRNA expression at the two-cell stage (Figure 4B) and significantly improved the cleavage rate and blastocyst rate of the injected embryos (Table 3).

### 2.4. Effects of PDCD6 siRNA Microinjection on the Transcriptome of Injected SCNT Embryos at the Two-Cell Stage

**Figure 5:**
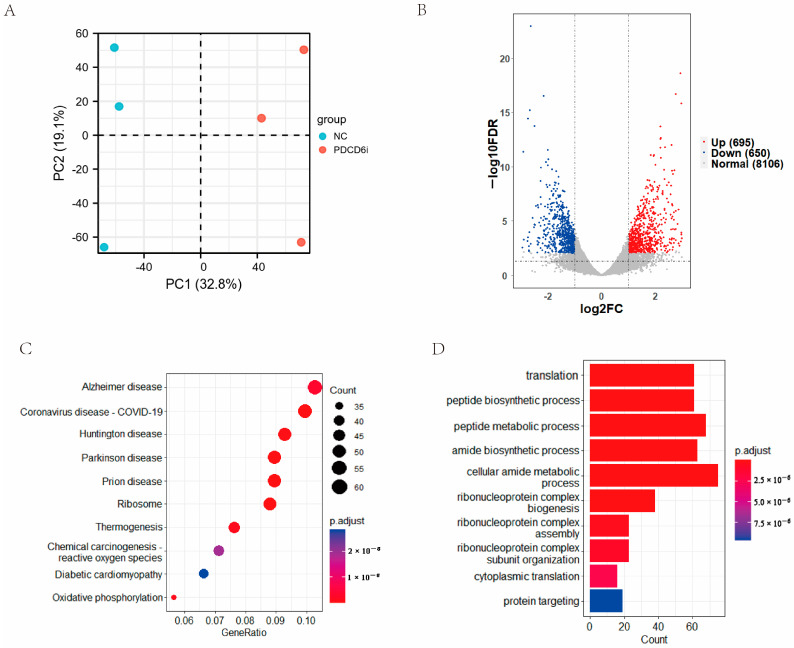
Transcriptomic analysis of two-cell stage SNCT embryos in the PDCD6i and NC groups. (**A**) PCA analysis of the NC and PDCD6i groups. (**B**) Volcano plot of the DEGs between the NC and PDCD6i groups. (**C**) Pathway enrichment analysis of the DEGs based on the KEGG database. The top 10 entries are shown. (**D**) Function enrichment analysis of the DEGs based on the GO database. The top 10 entries are shown.

To examine the effects of *PDCD6* knockdown on the global gene expression of injected cloned embryos, we collected two-cell stage knockdown embryos and control embryos for transcriptomic sequencing. The PCA results showed that three samples in the negative control (NC) group were obviously separated from the samples in the *PDCD6* siRNA (PDCD6i) group (Figure 5A). A total of 9451 annotated genes were detected by transcriptomic sequencing, which included 695 upregulated genes and 650 downregulated genes in the PDCD6i group compared with the NC group (Figure 5B). KEGG analysis showed that the differentially expressed genes (DEGs) were mainly enriched in several disease-related, ribosome, thermogenesis, and amide biosynthesis pathways, among others. (Figure 5C). GO analysis suggested that the DEGs were mainly enriched in translation, peptide biosynthesis, peptide metabolism, and amide biosynthesis (Figure 5D).

### 2.5. PDCD6 siRNA Microinjection Inhibited the Expression of Pro-Apoptotic Genes in Injected Cloned Embryos

**Figure 6:**
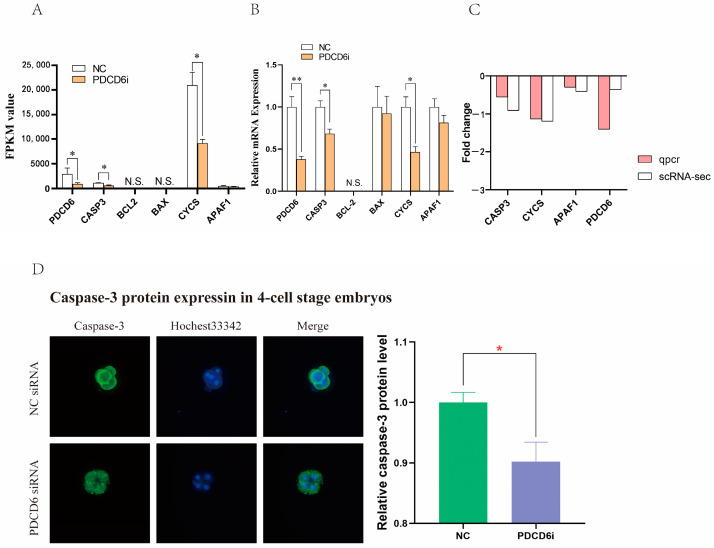
*PDCD6* siRNA microinjection inhibited the expression of pro-apoptotic genes in cloned porcine embryos. (**A**) The fragments per kilo base of transcript per million mapped fragments (FPKM) values of the apoptosis-related genes from the RNA sequencing results of the cloned porcine embryos. (**B**) Relative mRNA expression levels of the apoptosis-related genes in the cloned two-cell stage porcine embryos, as measured by qPCR. “*” and “**” represent a significant difference at *p* < 0.05 and *p* < 0.01, respectively; “NT” indicates non-detectable values. (**C**) RNA-sequencing and qPCR-measured fold change (PDCD6i vs. NC) in the mRNA level of apoptosis-related genes. (**D**) Comparison of CASP3 protein levels in cloned pig embryos in the NC and PDCD6i groups. This picture was taken by the Nikon fluorescent microscope with a magnification of 200×.

*PDCD6* participates in apoptosis by regulating the expression of *caspase-3* (*CASP3*), a pro-apoptotic gene. [29,30]. To study whether RNAi-mediated knockdown of *PDCD6* expression affected the expression of apoptosis-related genes in injected cloned porcine embryos, we extracted the fragments per kilo base of transcript per million mapped fragments (FPKM) values of six apoptosis-associated genes from the transcriptome sequencing results. The data demonstrated that the transcription levels of *PDCD6*, *CASP3* and its upstream gene *Cytochrome C* (*CYCS*) were significantly decreased in the PDCD6i group compared with the NC group (Figure 6A). These results were confirmed by quantitative polymerase chain transcription (qPCR) analysis (Figure 6B,C). To further investigate whether inhibition of PDCD6i expression via siRNA injection affected CASP3 protein abundance in injected cloned embryos, we compared the protein expression levels of CASP3 between the NC and PDCD6i groups. We found that CASP3 protein levels were significantly decreased in the PDCD6i group compared with the NC group, which was consistent with the transcriptomic sequencing and qPCR results (Figure 6D). Overall, these results indicated that *PDCD6* siRNA injection can reduce the mRNA and protein levels of pro-apoptotic CASP3 in cloned porcine embryos.

### 2.6. Intersection Analysis of the Microproteome and Transcriptome Results Identified Potential Factors That Regulate Cloned Porcine Embryo Development

The above results suggest that correcting the expression patterns of key regulators improved the developmental ability of pig SCNT embryos in the PDCD6i group. Similarly, the higher developmental potential seen in the NT-22b group was attributed to the relatively normal gene expression pattern in this group of embryos. Therefore, the DEGs that were identified by transcriptomics between the PDCD6i and NC groups at the two-cell stage may share similar factors with the DEPs identified by microproteomics between the NT22b and NT-211 groups at the same stage. These shared factors may be important regulators in cloned pig embryo development.

**Figure 7:**
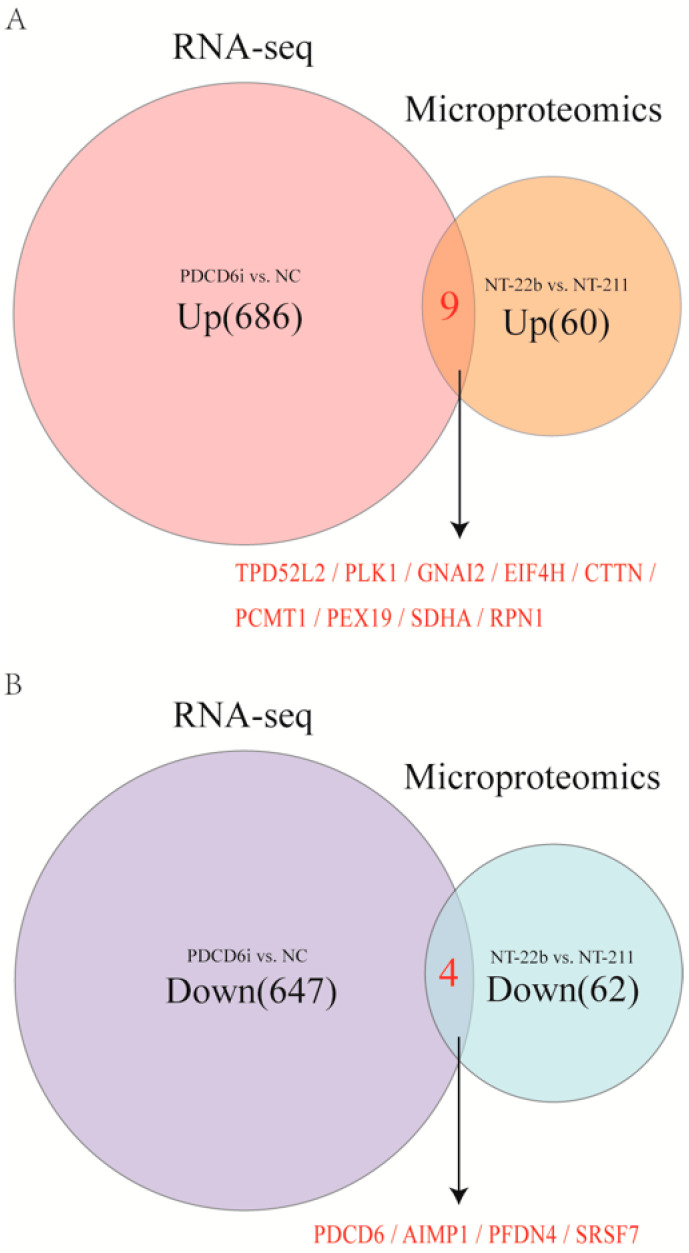
Discovery of potential factors regulating cloned porcine embryo development by intersection analysis of the microproteome and transcriptome (RNA-sequencing) results. (**A**) Intersection analysis of the upregulated genes and upregulated proteins in the PDCD6i and NC groups and the NT-22b and NT-211 groups, respectively. (**B**) Intersection analysis of the downregulated genes and downregulated proteins in the PDCD6i and NC groups and the NT-22b and NT-211 groups, respectively.

To analyze these shared factors, we conducted an intersection analysis of the upregulated DEGs and upregulated DEPs in the PDCD6i vs. NC groups and the NT22b vs. NT-211 groups, respectively, as well as the downregulated DEGs and downregulated DEPs in the PDCD6i vs. NC groups and the NT22b vs. NT-211 groups, respectively. The results indicated that the two upregulated gene (protein) sets share nine factors, specifically *TPD52L2*, *PLK1*, *GNA12*, *EIF4H*, *CTTN*, *PCMT1*, *PEX19*, *SDHA*, and *RPN1*(Figure 7A), while the two downregulated gene (protein) sets share four factors, specifically *PDCD6*, *AIMP1*, *PFDN4*, and *SRSF7* (Figure 7B).

### 2.7. PLK1 mRNA Injection Significantly Increased the Cleavage Rate of Cloned Porcine Embryos

**Figure 8:**
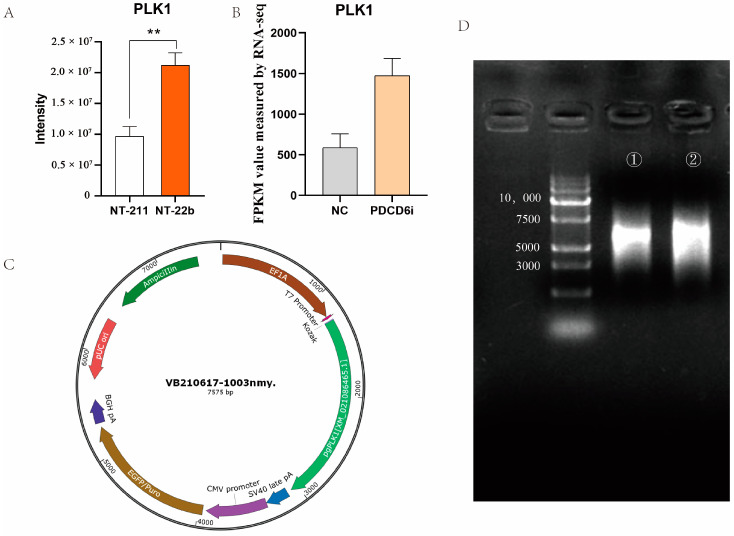
Overexpression of *PLK1* in cloned pig embryos. (**A**) PLK1 protein abundance in the NT-211 and NT-22b groups, as measured by microproteomics. “**” represent a significant difference at *p* < 0.01. (**B**) *PLK1* mRNA levels in the NC and PDCD6i groups, as measured by transcriptomics. (**C**) *PLK1* expression plasmid map. (**D**) *PLK1* mRNA produced via in vitro transcription. ① and ② denotes the electrophoresis analysis of *PLK1* mRNA after 3′-capping and 5′-tailing, respectively.

We identified 13 intersection genes (proteins) from the transcriptomic and microproteomic data. Among the DEPs identified by microproteomics, *PLK1* was involved in two enriched KEGG pathways, the cell cycle, and the progesterone-mediated oocyte maturation pathways (Figure 3B,C). *PLK1* has previously been identified as essential to the development of cloned and fertilization-derived embryos [31,32,33]. Furthermore, the PLK1 protein expression level was significantly lower in the NT-211 group compared to the NT-22b group (Figure 8A), and the PLK1 mRNA expression level was significantly lower in the NC group in comparison to the PDCD6i group (Figure 8B), suggesting that insufficient PLK1 expression may be responsible for the developmental arrest of NT-211 cloned embryos.

To test whether *PLK1* overexpression can enhance developmental competence in cloned embryos, we constructed a *PLK1* expression plasmid (Figure 8C) that produced mature *PLK1* mRNA via in vitro transcription (Figure 8D). We microinjected different concentrations of *PLK1* mRNA into cloned porcine embryos and found that 1000 ng/μL of *PLK1* mRNA significantly increased the cleavage rate and blastocyst rate of injected SCNT embryos (Table 4). These results implied that *PLK1* helps regulate cloned porcine embryo development.

## 3. Discussion

Previous studies utilized microproteomics to identify 348 and 667 proteins in mouse and sheep pre-implantation embryos, respectively [34,35]. Using a similar microproteomics technology, we identified 2523 proteins in the blastomere samples of pig SCNT embryos. Although the number of proteins detected in this study is much higher than in previous studies with other animal embryos, it is still lower than the number of proteins identified by routine proteomics. Routine proteonomics typically identifies 3500–5000 proteins in pig tissue or cell samples [36,37,38,39,40]. Therefore, the microproteomics method used in this study still needs to be optimized or modified to improve its ability to detect low abundance proteins in analyzed samples. Furthermore, the number of annotated proteins in the pig database is far lower than that in the mouse and human databases [41]. As such, approximately one-third of the peptides identified in this study were unknown and remain to be annotated in the future.

*PDCD6*, or *ALG-2*, is a known pro-apoptotic gene [22,24,42,43]. In this study, we found that PDCD6 protein levels were much higher in the NT-211 group compared with the NT-22b group, which suggested that high PDCD6 protein levels in NT-211 cloned embryos may induce high levels of apoptosis and inhibit cloned NT-211 embryo development. *PDCD6* knockdown through *PDCD6* siRNA injection significantly increased blastocyst rates in cloned porcine embryos and significantly reduced the mRNA and protein expression levels of *CASP3*, another apoptosis-promoting gene. This suggests that *PDCD6* is involved in the regulation of *CASP3* expression in cloned porcine embryos. Similar results have been reported in literature [29,30]. Our results also showed that expression levels of *CYCS*, another apoptosis-related gene, in cloned *PDCD6* knockdown embryos were significantly lower than in the control group. The *CYCS* gene encodes a pro-apoptotic factor that is commonly involved in the respiratory chain and in electron transfer [44]. Once released into the cytosol, *CYCS* becomes a vital part of the apoptosis pathway by activating CASP3 [44,45,46]. Therefore, *PDCD6* may affect the expression of CASP3 by regulating *CYCS* expression.

*PLK1* acts on the cell cycle and progesterone-mediated oocyte maturation pathways and is the main regulator of early embryo mitosis. [31,33,47,48]. In the present study, injection of 1000 ng/mL of *PLK1* mRNA into cloned porcine embryos significantly increased the cleavage rate and tended to enhance the blastocyst rate of the embryos. This indicates that *PLK1* participates in the regulation of cloned porcine embryo development. Nevertheless, the blastocyst rate of the *PLK1* overexpression group was not significantly higher than that of the control group, which might be related to the insufficient amount of *PLK1* mRNA that was injected into the embryos. However, 1000 ng/mL was the highest possible concentration that we could prepare for *PLK1* mRNA. Future studies can determine whether injection of higher concentrations of *PLK1* mRNA can result in higher rates of cloned porcine embryo development than that observed in this study.

In this study, embryo biopsy in combination with microproteomics was employed to identify the potential molecules responsible for the arrest at the two-cell stage in cloned porcine embryos. This approach can also be used to identify the important factors that cause the developmental defects at the four-cell stage of pig SCNT embryos, as has been reported [15]. A group of potentially critical regulators of cloned porcine embryo development has been identified and only two, *PDCD6* and *PLK1*, were tested in this study. In the future, other factors in the gene (protein) sets identified in this study can be examined to determine whether they also play important roles in the development of SCNT pig embryos.

## 4. Materials and Methods

### 4.1. In Vitro Oocyte Maturation

The ovaries of 6- to 7-month-old gilts were purchased from Panyu Slaughterhouse in Guangzhou. These ovaries were delivered to our laboratory in a 0.9% (wt/vol) sodium chloride solution supplemented with penicillin-G (100 IU/mL) and streptomycin sulfate (100 mg/L). The ovaries were delivered at 30–35 °C within 3 h of removal from the gilts. Using a 10 mL syringe with an 18-G needle, follicular fluid was collected from 3–8-mm-sized follicles and placed in centrifuge tubes. The cumulus oocyte complexes (COCs) in the follicular fluid were washed with Dulbecco’s phosphate-buffered saline (DPBS) (GIBCO, Carlsbad, CA, USA) four times. The complexes with dense cumulus cells and uniform cytoplasms were selected and cultured with M199 complete medium (Sigma-Aldrich, St. Louis, MO, USA) under a humid atmosphere of 38.5 °C and 5% CO_2_ for 42–44 h. The cumulus cells of mature COCs around the oocytes were removed by gentle blowing with DPBS containing 1 mg/mL hyaluronidase in a pipette. Oocytes with intact cell membranes and first polar bodies were selected under a stereomicroscope for subsequent SCNT.

### 4.2. Cell Culture

Primary ear skin fibroblasts were isolated by the tissue block attachment method. Primary fibroblasts were cultured in complete medium for 3–4 days to reach 80–90% confluency prior to SCNT. Adherent cells were treated with trypsin for 1 min then used for SCNT.

### 4.3. SCNT

In vitro mature oocytes and a small number of donor cells were placed in T2 medium (TCM-199 plus 2% fetal bovine serum) containing 7.5 μg/mL cytochalasin B. An enucleated pipette (Lingen Precision Medical Products Co., Ltd., Shanghai, China) with an inner diameter of 17 μm was inserted into the oocytes to aspirate the first polar body along with adjacent cytoplasm containing genomic DNA. Enucleated oocytes were stained with 1 g/mL Hoechst 33,342 and examined under ultraviolet light irradiation. Only fully enucleated oocytes were selected for SCNT. After digestion with 0.25% trypsin, a single donor cell with round and slightly burr-like shape was injected into the perivitelline space of each enucleated oocyte by the injection needle. The reconstructed embryos were cultured in pig zygote medium-3 (PZM-3) for 1 h and activated in the fusion solution through two direct current pulses of 150 V/mm for 50 ms. Activated embryos were cultured in an incubator with 38.5 °C, saturated humidity, and 5% CO_2_.

### 4.4. Blastomere Isolation from Cloned Embryos

At 4 h post-activation, the SCNT embryos were treated with tyrode solution (Sigma-Aldrich, St. Louis, MO, USA) and 0.5% streptomycin (Sigma-Aldrich, St. Louis, MO, USA) to remove the zona pellucida. The embryos were then cultured in the microwells of a culture plate with one embryo in each well. At 24 h (1 day) post-activation, one blastomere was isolated from each two-cell stage embryo and frozen for later microproteomic analysis. The remaining blastomeres in each embryo were cultured to observe their developmental status at 48 h (2 days) and 168 h (7 days) post-activation.

### 4.5. Microproteomic Analysis

Auxiliary library building and microproteome sequencing were carried out by Shenzhen Huada Gene Co., Ltd. (Shenzhen, China) The primary pig skin fibroblast and pig ovarian tissue samples were used for auxiliary library building. An appropriate amount of lysis buffer was added to the blastomere samples for protein extraction, and all blastomeres were vibrated and sonicated for lysis. The blastomere proteins were precipitated and centrifugated, and the resulting supernatant was carefully collected with a pipette and treated with protein alkylation. For the qualified samples, 50 μg of protein solution were taken from each group for trypsin enzymolysis. The samples were then desalinized, and 20 components were separated with high pH RP. The peptides obtained from this process were injected into a Thermo Ultimate 3000 UHPLC (Thermo Fisher Scientific, Waltham, MA, USA) configured with a trap column containing C18. The samples were enriched and desalted in the trap column and then separated in series through the C18 column at a flow rate of 500 nL/min. The separated fractions were analyzed in data-dependent acquisition mode using a nanoESI source. The data was then entered into the Orbitrap Fusion™ Lumos™ Tribrid™ (Thermo FisherScientific, Waltham, MA, USA) tandem mass spectrometer. The final microproteome samples were identified and quantified using an Andromeda engine integrated with MaxQuant software, and the measured peptide sequences were compared with the Uniport database (https://www.uniprot.org/, accessed on 2 April 2021) to identify the corresponding protein types.

### 4.6. PDCD6 siRNA Design, Synthesis, and Transfection

According to the mRNA sequences of the porcine *PDCD6* gene, three siRNA duplexes were designed, chemically modified, and synthesized by GenePharma Co., Ltd. (Shanghai, China). The synthesized *PDCD6* siRNAs were then transfected into the porcine fibroblasts with the Lipofectamine RNAi Max Reagent (Invitrogen, Carlsbad, CA, USA) according to the manufacturer’s instructions. The sequence information for 3 *PDCD6* siRNAs is as follows:

siRNA-155 (forward): 5′-GCUUCCUGUGGAACGUCUUTT-3′;
siRNA-155 (reverse): 5′-AAGACGUUCCACAGGAAGCTT-3′;
siRNA-204 (forward): 5′-GAUAUCGGACAACGAGCUUTT-3′;
siRNA-204 (reverse): 5′-AAGCUCGUUGUCCGAUAUCTT-3′;
siRNA-314 (forward): 5′-GCGUGAAUUUCAGCGAGUUTT-3′;
siRNA-314 (reverse): 5′-AACUCGCUGAAAUUCACGCTT-3′;
NC-siRNA (forward): 5′-UUCUCCGAACGUGUCACGUTT-3′;
NC-siRNA (reverse): 5′-ACGUGACACGUUCGGAGAATT-3′.

### 4.7. PLK1 mRNA Synthesis

The *PLK1* expression plasmid was obtained by inserting the coding sequences of the synthetic porcine *PLK1* gene (GenBank no. XM_021086465.1) from multiple cloning sites on the pRP [Exp]-EGFP/Puro-EF1A/T7 vector. The linearized *PLK1* expression plasmid was used as a template to produce *PLK1* mRNA with an in vitro transcription kit (mMESSAGE mMACHINE^®^ T7 Ultra Kit; Thermo Fisher Scientific, Waltham, MA, USA). The produced *PLK1* mRNA was analyzed by electrophoresis.

### 4.8. Microinjection

Microinjection was performed using a micropipette driven by a Piezo (Eppendorf, Hamburg, Germany) 2 h after activation of the SCNT embryos. Ten pL of PDCD6 siRNA or PLK1 mRNA was microinjected into each cloned pig embryo. Control embryos were injected with the same volume of NC siRNA or NC mRNA.

### 4.9. Transcriptome Sequencing

Two-cell stage injected embryos were collected 24 h after siRNA injection. Twenty to thirty embryos from each group were collected and placed in a 1-mL tube which contained 6 µL of lysis buffer with an RNase inhibitor. Complementary DNA (cDNA) was generated and amplified for sequencing based on the smartseq2 method. The amplified double-stranded cDNA was spliced by Tn5 transposition enzyme digestion, PCR amplification, and magnetic bead purification. The overall quality of the initial cells was detected by the Agilent 2100 Bioanalyzer (Santa Clara, CA, USA). The aggregated library preparations were sequenced on the NovaSeq 6000 (Illumina, San Diego, CA, USA) platform, which generated 150 paired-end readings.

### 4.10. qPCR Analysis

Total RNAs were extracted from the porcine fibroblasts and microinjected embryos using the RNeasyPlus Micro Kit (Qiagen, Hilden, Germany). cDNA was then synthesized using a PrimeScirpt RT Reagent Kit with gDNA Eraser (Takara Bio Inc., Kusatsu, Japan). β-actin was used as an endogenous control gene. For qPCR, SYBR Green Real-time PCR Master Mix reagents (Toyobo Co., Ltd., Osaka, Japan) and sense and antisense primers (200 nM for each gene) were used. The PCR reactions were carried out in a QuantStudio 7 Flex system (Thermo Fisher Scientific, Waltham, MA, USA). The primer sequences were as follows:

PDCD6 (forward): 5′-AAGACAGGAGCGGCGTGATAT-3′;
PDCD6 (reverse): 5′-CGTTCTGCCAGTCGGTGATG-3′;
CASP3 (forward): 5′-GGACTGCTGTAGAACTCTAACTGG-3′;
CASP3 (reverse): 5′-CAAGAAGTCTGCCTCAACTGGTAT-3′;
BCL2 (forward): 5′-GTGTGTGGAGAGCGTCAACC-3′;
BCL2 (reverse): 5′-CCTTCAGAGACAGCCAGGAGAA-3′;
BAX (forward): 5′-TCTACCAAGAAGTTGAGCGAGTGT-3′;
BAX (reverse): 5′-CCAGTTGAAGTTGCCGTCAGC-3′;
CYCS (forward): 5′-CTGCGAGTGGTGGCTTGTCT-3′;
CYCS (reverse): 5′-CAGTCTTGTGTTTGCCTCCCTTT-3′;
APAF1 (forward): 5′-CGACTGGAGATGACAACGGAGAA-3′;
APAF1 (reverse): 5′-ACTAAGACTGGAGCACACGAATGA-3′;
β-actin (forward): 5′-CCACGAGACCACCTTCAACTC-3′;
β-actin (reverse): 5′-TGATCTCCTTCTGCATCCTGT-3′;

### 4.11. Immunofluorescence

The collected cloned embryos were fixed with a 4% paraformaldehyde fixative (Beyotime Biotechnology, Shanghai, China) for 15 min, washed thrice with PBS, penetrated by 0.5% TritonX-100 (Beyotime Biotechnology) for 20 min and sealed with an immunofluorescence sealing solution (Beyotime Biotechnology) at room temperature for 30 min. The embryos were incubated with CSP3-antibody (1:100; A0214; ABclonal; Wuhan, China) and goat anti-rabbit IgG H&L (1: 200; ab150077; Abcam, England) at room temperature for 1 h and at 4 ℃ for 12 h, respectively. After incubation, the embryos were washed thrice with PBS, stained with Hochest 33,342 (10 ug/mL; Yeasen Biotechnology Co., Ltd., Shanghai, China) for ten min, then washed and observed under the Nikon Eclipse Ti-s microscope (Nikon Instruments, Shanghai, China). Images were captured with the Nis Elements BR software (Nikon Instruments, Shanghai, China).

### 4.12. Statistical Analysis

GraphPad Prism 8.0 (GraphPad, San Diego, CA, USA) was used for statistical analysis. Differences in values from more than two groups were determined by one-way analysis of variance. Differences between two groups were analyzed by Student’s *t*-test.

## 5. Conclusions

We used embryo biopsy combined with microproteomics to identify a group of potential key factors that regulate cloned porcine embryo development. Two factors, *PDCD6* and *PLK1*, were verified to play important roles in the development of pig SCNT embryos. In particular, *PDCD6* knockdown and *PLK1* overexpression enhanced the developmental competence of treated pig SCNT embryos. This study helped elucidate the molecular mechanisms underlying developmental arrest in cloned porcine embryos, as well as developed new methods for improving pig cloning efficiency.

## Figures and Tables

**Table 1:** The developmental fates of SCNT pig embryos after isolation of a blastomere at the two-cell stage.

	Manipulated Two-Cell Stage Embryos	Arrested at the Two-Cell Stage	Arrested at the Four-Cell Stage	Developed to the Blastocyst Stage	Arrested at Other Stages
Total number of embryos	335	165	58	66	46
Proportion(%)	100%	49.25%	17.31%	19.70%	13.73%
Group name	-	NT-211	NT-222	NT-22b	-

**Table 2:** Number of peptides and proteins identified by microproteomics.

Samples	Number of Peptides	Number of Proteins
NT211_1	2296	1135
NT211_2	1867	823
NT211_3	2039	936
NT211_4	2120	963
NT211_5	1897	840
NT211_6	2366	1075
NT222_1	2366	1075
NT222_2	1632	740
NT222_3	2248	1076
NT222_4	1836	841
NT222_5	2471	1156
NT222_6	1294	648
NT22b_1	2470	1176
NT22b_2	2496	1186
NT22b_3	1883	955
NT22b_4	1971	935
NT22b_5	2163	933
NT22b_6	1554	825

**Table 3:** The effects of *PDCD6* siRNA microinjection on the in vitro developmental efficiency of pig SCNT embryos.

Total Number of Injected SCNT Embryos	Concentration of *PDCD6* siRNA	Repetition Numbers	Cleavage Rate (%)	Blastocyst Rate (%)	Number of Cells per Blastocyst
120	0 μM	4	65.84 ± 9.18	11.67 ± 1.92	42.13 ± 7.40
126	5 μM	4	71.40 ± 4.84	11.89 ± 1.49	46.50 ± 6.63
119	10 μM	4	75.58 ± 4.68	14.43 ± 3.31	48.63 ± 7.48
114	20 μM	4	79.13 ± 5.65 *	17.55 ± 2.94 *	47.75 ± 5.00

Values labeled with * means they are significantly different at *p* < 0.05 from that of the NC group in the same column.

**Table 4:** The effects of *PLK1* mRNA microinjection on the in vitro developmental efficiency of SCNT pig embryos.

Total Number of Injected SCNT Embryos	Concentration of *PLK1* mRNA	Repetition Numbers	Cleavage Rate (%)	Blastocyst Rate (%)	Number of Cells per Blastocyst
116	0 ng/μL	3	63.22 ± 1.99	11.49 ± 5.27	44.83 ± 9.87
115	100 ng/μL	3	54.56 ± 12.52	10.40 ± 3.45	47.83 ± 10.63
108	500 ng/μL	3	66.66 ± 9.81	8.64 ± 4.28	43.83 ± 7.14
113	1000 ng/μL	3	77.38 ± 5.45 *	15.48 ± 2.06	48.67 ± 9.73

Values labeled with * means they are significantly different at *p* < 0.05 from that of the NC group in the same column.

## Data Availability

The corresponding author can provide access to the datasets upon request.

## Abbreviation

APAF1	apoptotic protease activating factor 1
BCL-2	B-cell lymphoma 2
CASP3	Caspase-3
cDNA	Complementary DNA
CHIP-seq	Chromatin immunoprecipitation sequence
COCs	Cumulus oocyte complexes
CYCS	Cytochrome
DEGs	Differentially expressed genes
DEPs	Differentially expressed proteins
DNA	Deoxyribonucleic acid
DPBS	Cumulus oocyte complexes
DPBS	Dulbecco’s phosphate-buffered saline
FPKM	Fragments per kilobase of exon model per million mapped fragments
GO	Gene ontology
KEGG	Kyoto Encyclopedia of Genes and Genomes
mRNA	Messenger RNA
PCA	Principal component analysis
PDCD6	Programmed cell death protein 6
PDCD6i	PDCD6 siRNA
PLK1	Polo-like kinase 1
PZM-3	Porcine zygote medium 3
qPCR	Quantitative real-time PCR
RNA	Ribonucleic acid
SCNT	Somatic cell nuclear
siRNA	Small interfering RNA
μL	Microlitre
μM	Micromole/L
